# Crystal structures of two 1,3-thia­zolidin-4-one derivatives featuring sulfide and sulfone functional groups

**DOI:** 10.1107/S2056989018015098

**Published:** 2018-11-06

**Authors:** Hemant P. Yennawar, Lee J. Silverberg, Kevin Cannon, Deepa Gandla, Sandeep K. Kondaveeti, Michael J. Zdilla, Ahmed Nuriye

**Affiliations:** a Department of Biochemistry and Molecular Biology, The Pennsylvania State University, University Park, Pennsylvania, 16802, USA; b Department of Chemistry, The Pennsylvania State University, Schuylkill Campus, 200 University Drive, Schuylkill Haven, Pennsylvania, 17972, USA; c Department of Chemistry, The Pennsylvania State University, Abington College, 1600 Woodland Road, Abington, Pennsylvania, 19001, USA; d Department of Chemistry, Temple University, 1901 North 13th Street, Philadelphia, Pennsylvania, 19122, USA

**Keywords:** crystal structure, thia­zolidin-4-one, thia­zolidinone, envelope pucker, chair conformation

## Abstract

The crystal structures of two closely related compounds, 1-cyclo­hexyl-2-(2-nitro­phen­yl)-1,3-thia­zolidin-4-one, C_15_H_18_N_2_O_3_S, **(1)** and 1-cyclo­hexyl-2-(2-nitro­phen­yl)-1,3-thia­zolidin-4-one 1,1-dioxide, C_15_H_18_N_2_O_5_S, **(2)**, are presented. These compounds are comprised of three types of rings: thia­zolidinone, nitrophenyl and cyclo­hexyl. In both structures, the rings are close to mutually perpendicular, with inter­planar dihedral angles greater than 80° in each case. The thia­zol­idinone rings in both structures exhibit envelope puckering with the S atom as flap and the cyclo­hexyl rings are in their expected chair conformations. The two structures superpose fairly well, except for the orientation of the nitro groups with respect to their host phenyl ring, with a difference of about 10° between **1** and **2**. The extended structure of **1** has two kinds of weak C—H⋯O inter­actions, giving rise to a closed ring formation involving three symmetry-related mol­ecules. Structure **2** has four C—H⋯O inter­actions, two of which are exclusively between symmetry-related thia­zolidinone dioxide moieties and have a parallel ‘give-and-take-fashion’ counterpart. In the other two inter­actions, the nitrophenyl ring and the cyclo­hexane ring each offer an H atom to the two O atoms on the sulfone group. Additionally, a C—H⋯π inter­action between a C—H group of the cyclo­hexane ring and the nitrophenyl ring of an adjacent mol­ecule helps to consolidate the structure.

## Abstract 2

The closely related title compounds are comprised of three types of rings: thia­zolidinone, nitrophenyl and cyclo­hexyl. In both structures, the rings are close to mutually perpendicular, with inter­planar dihedral angles greater than 80° in each case.

## Chemical context   

The title compounds were synthesized as a part of our ongoing work on the synthesis of new types of 2,3-disubstituted 1,3-thia­zolidin-4-ones. We have reported the crystal structures of a number of these compounds before (Nuriye *et al.*, 2018 ▸; Yennawar *et al.*, 2015 ▸). These compounds are synthesized by a tandem nucleophilic addition-carbonyl condensation of thio­glycolic acid with the desired *in situ*-generated imine. The variation in substitution pattern is set during the synthesis of the imine where alkyl or aryl amines are condensed with an aldehyde (Surrey, 1947 ▸; von Erlenmeyer & Oberlin, 1947 ▸). In addition, the S atom in the thia­zolidinone ring can be oxidized to the sulfoxide or the sulfone to produce structures with different properties. Thia­zolidinones have well documented biological activity (Thakare *et al.*, 2018 ▸; Brown, 1961 ▸; Abdel Rahman *et al.*, 1990 ▸; Joshi *et al.*, 2014 ▸; Suryawanshi *et al.*, 2017 ▸; Kaushal & Kaur, 2016 ▸; Kumar *et al.*, 2015 ▸; Tripathi *et al.*, 2014 ▸; Jain *et al.*, 2012 ▸; Abhinit *et al.* 2009 ▸; Hamama *et al.*, 2008 ▸; Singh *et al.*, 1981 ▸). The synthesis and characterization of these compounds could be valuable in investigations for the practical applications of their activities. To the best of our knowledge, only two crystal structures of thia­zolidinone sulfones have been reported in the literature (Orsini *et al.*, 1995 ▸; Glasl *et al.*, 1997 ▸). The compounds presented in this paper both feature an *ortho*-nitro­phenyl ring at position 2 and a cyclo­hexane ring at the 3-position of the thia­zolidinone ring. Compound **1** is a sulfide, while compound **2** contains a fully oxidized sulfone functional group.
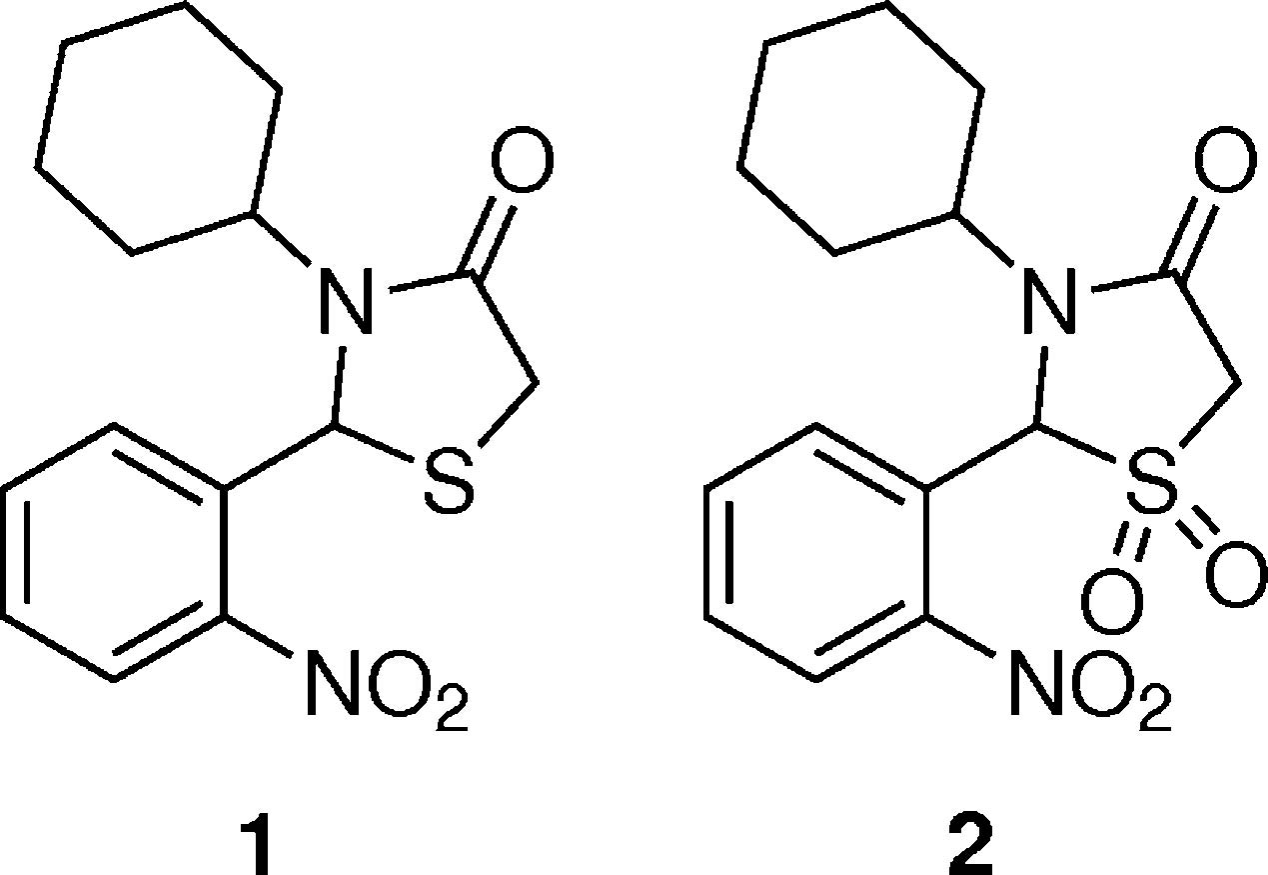


## Structural commentary   

**Figure 1:**
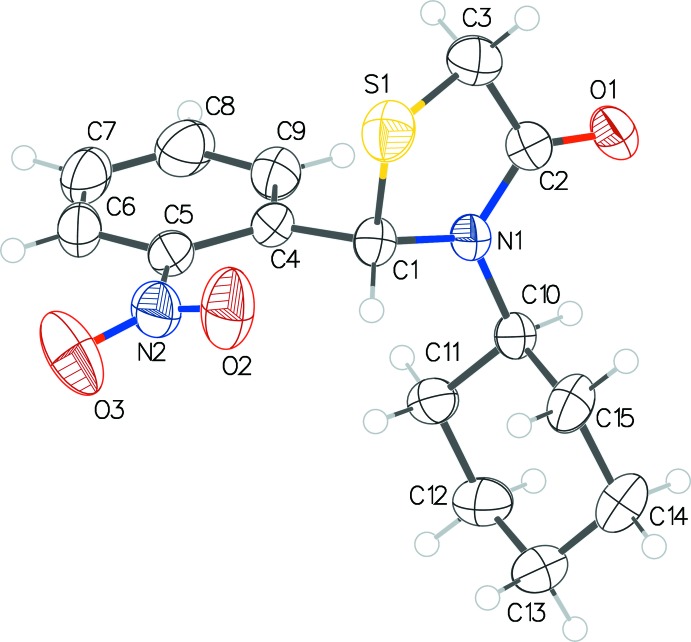
The mol­ecular structure of **1** with displacement ellipsoids drawn at the 50% probability level.

**Figure 3:**
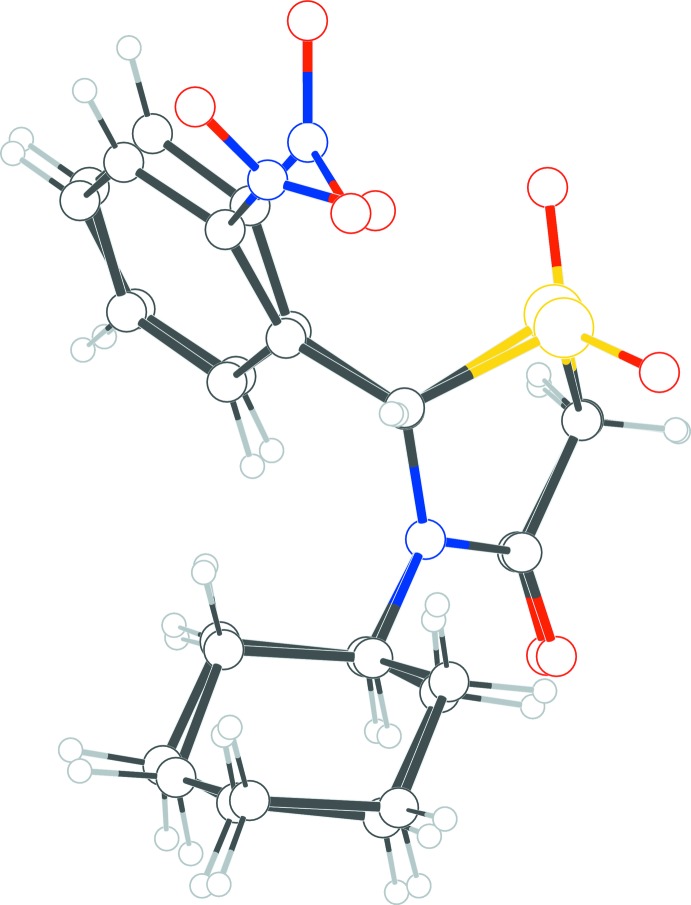
Overlay image of the two title mol­ecules showing the difference in the orientation of the nitro group with respect to the nitrophenyl ring plane.

Compound **2** is the dioxide version of **1**, both comprising of three types of rings, a thia­zolidinone (*A*), a nitrophenyl (*B*) and a cyclo­hexyl (*C*) ring. In each structure, the inter­planar dihedral angles between the three pairs of rings are close to orthogonal, with values of (in ascending order) *A*/*C* = 84.04 (9), *B*/*C* = 84.98 (10) and *A*/*B* = 85.85 (9)°. The corres­ponding data for **2** span a slightly wider range: *B*/*C* = 80.74 (6), *A*/*B* = 83.12 (6) and *A*/*C* = 87.96 (6)° (Figs. 1 ▸ and 2 ▸). In both structures, the thia­zolidinone rings exhibit an envelope pucker conformation with the sulfur atom as a flap. The cyclo­hexyl rings are in the most stable chair conformation in both structures. An overlay of the two structures (Fig. 3 ▸) shows that they overlap well. Fig. 3 ▸ also shows that the nitro group plane in **2** is twisted further away by *ca* 10° from the nitrophenyl ring plane as compared to that in **1**; the dihedral angles between the nitro group plane and the host phenyl ring plane were found to be 18.3 (5)° in **1** and 28.3 (5)° in **2**.

Looking at the thia­zolidinone ring systems, the C1—N1 and C1—S1 bond lengths are 1.438 (3) and 1.839 (3) Å, respectively, for structure **1** and 1.4527 (13) and 1.8382 (12) Å for structure **2**. The N—C—S bond angle is found to be 105.22 (12)° in structure **1** and 101.36 (7)° in structure **2** indicating a compression of the N—C—S bond angle going from the sulfide to the sulfone. Bond length and angle values in the thia­zolidinone ring of the sulfide appear to be typical and match data that we have previously reported (Nuriye *et al.*, 2018 ▸). Although structural data for the sulfone are scarce, the data reported by Orsini *et al.* (1995 ▸) matches our findings.

## Supra­molecular features   

**Figure 4:**
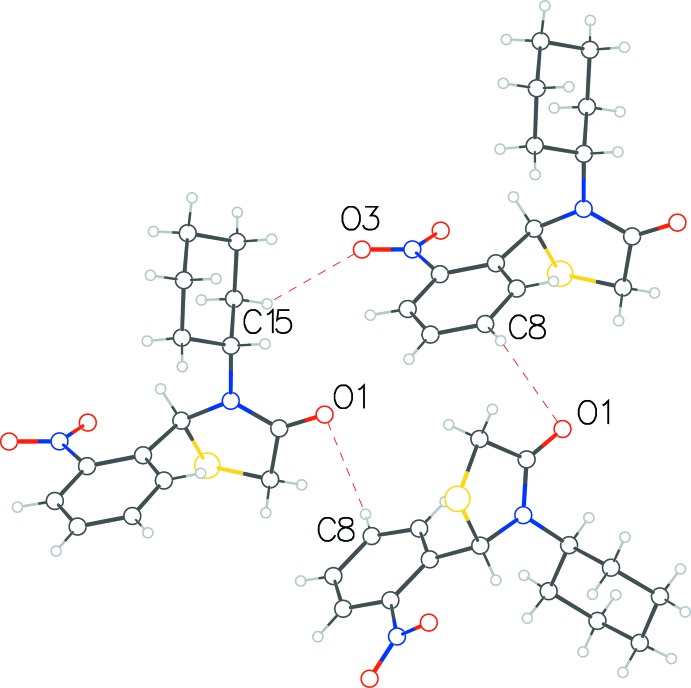
Hydrogen-bond inter­actions between three symmetry-related mol­ecules of **1** forming a closed-ring system.

**Figure 5:**
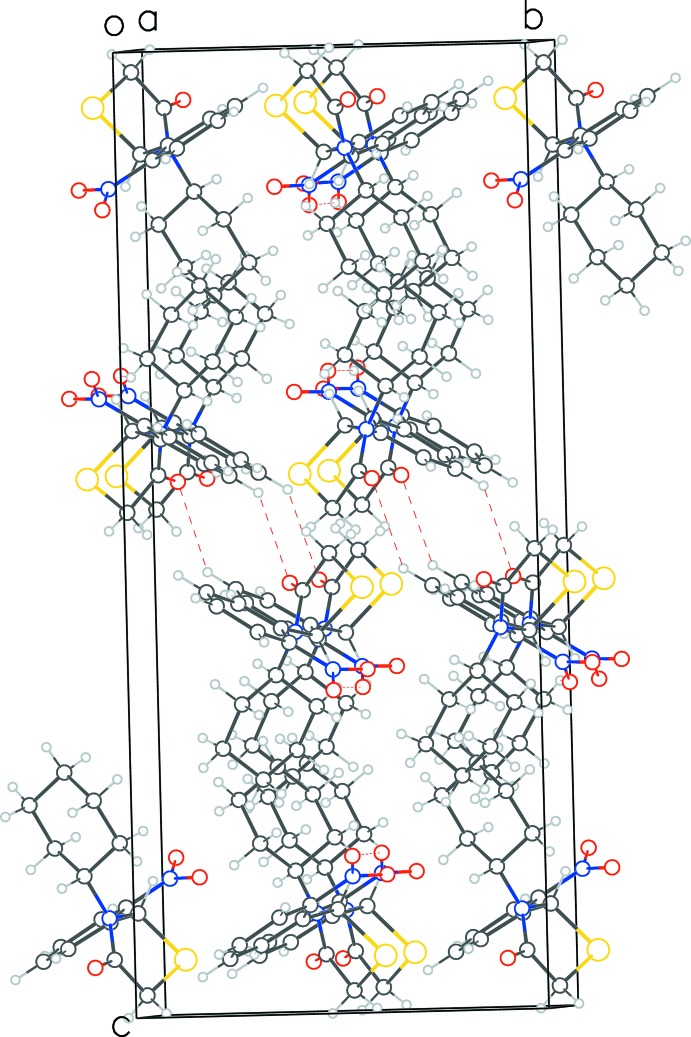
View down the *a* axis of the packing of **1**. The layering of mol­ecules in the (020) plane as well as the alternating pattern of hydro­phobic and hydro­philic regions perpendicular to *c* axis can be seen.

In structure **1**, two weak C—H⋯O type inter­actions (Table 1 ▸) result in a closed-ring formation of three symmetry-related mol­ecules (Fig. 4 ▸). One of the nitrophenyl-ring carbon atoms donates its H atom to the oxygen atom on the thia­zolidinone ring of a neighboring mol­ecule [C8⋯O1 = 3.411 (5) Å, C—H⋯O = 140°], which then inter­acts with a third symmetry-related mol­ecule through a *symmetry-equivalent* contact. Finally, this third mol­ecule donates one of its cyclo­hexane protons to the nitro­phenyl oxygen atom of the first mol­ecule [C15⋯O3 = 3.437 (5) Å, 138°], thus completing the three-mol­ecule ring arrangement. In the extended structure, the mol­ecules arrange themselves in distinct layers in (020) planes. Perpendicular to *c*, the longest axis, there is an alternating pattern of hydro­phobic and hydro­philic surfaces of the mol­ecules, as is evident in the packing diagram (Fig. 5 ▸).

**Figure 6:**
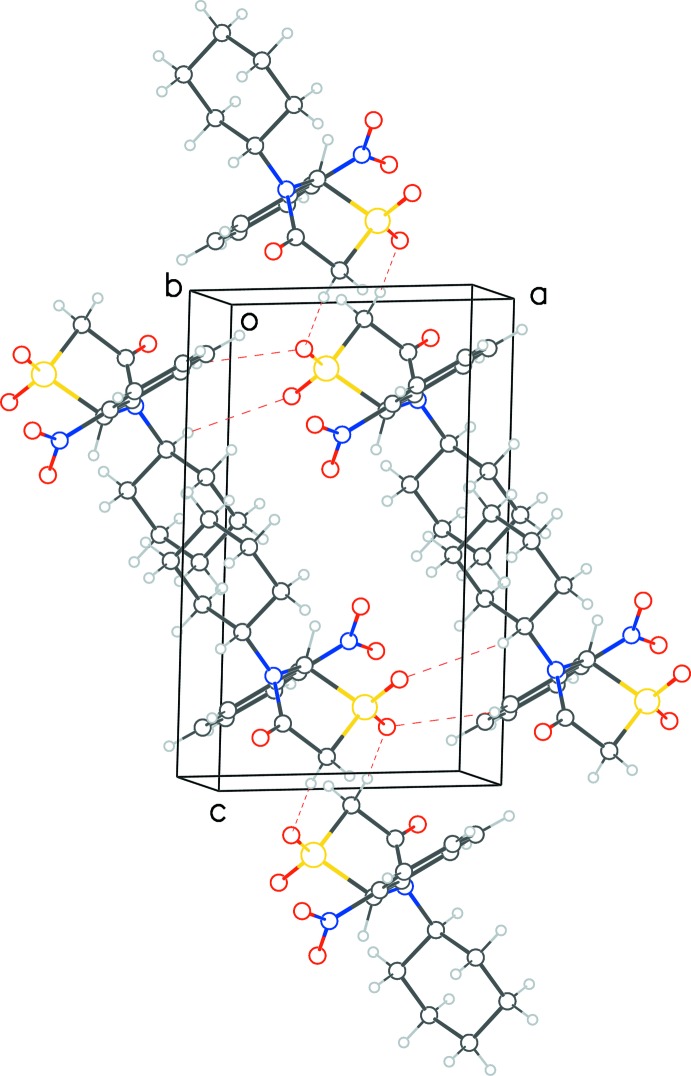
View down the *b* axis of the packing arrangement of **2**. The layering of mol­ecules in the (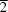
02) plane as well as the alternating pattern of hydro­phobic and hydro­philic regions perpendicular to the *c* axis can be seen.

In structure **2**, we observe four C—H⋯O type inter­actions (Table 2 ▸). Two of these involve the thia­zolidinone dioxide moieties exclusively and have parallel ‘give-and-take’ type counterparts [C⋯O = 3.4594 (16) Å, 161° and 3.3068 (16) Å, 157°], forming continuous chains propagating along the *b-*axis direction. The remaining two inter­actions are weaker and involve the carbon atoms of nitrophenyl rings and cyclo­hexane rings of one mol­ecule offering protons to the oxygen pair of the dioxide group [C9⋯O1 3.5144 (16) Å, 132.6° and 3.4381 (16) Å, 129°] of a symmetry-related mol­ecule. Similar to packing of **1**, the mol­ecules are arranged in distinct layers but this time in (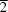
02) planes. Also seen is the alternating pattern of hydro­phobic and hydro­philic surfaces perpendic­ular to the *c*-axis direction (Fig. 6 ▸).

## Synthesis and crystallization   

**1-Cyclo­hexyl-2-(2-nitro­phen­yl)-1,3-thia­zolidin-4-one:** Following the reported method (Cannon *et al.*, 2015 ▸), 2-nitro­benzaldehyde (0.725 g, 4.80 mmol) was dissolved in CH_2_Cl_2_ (20 ml) and anhydrous MgSO_4_ (3.0 g) and cyclo­hexyamine (0.5 g, 5 mmol) were added sequentially and stirred for 4 h at r.t. under nitro­gen. The MgSO_4_ was filtered off and the reaction was concentrated *in vacuo* to give 0.9826 g of an orange oil, which solidified upon sitting in a freezer and remained solid upon warming up to room temperature.

The crude imine was resuspended in toluene (25 ml) and thio­glycolic acid (0.55 g, 6.0 mmol) was added and the reaction was heated at reflux for 1.5 h with a Dean–Stark trap attached. The reaction was then cooled to room temperature and washed with aqueous NaHCO_3_ (2 × 35 ml). The combined organic layers were dried over anhydrous Na_2_SO_4_, filtered, and concentrated under reduced pressure to give an orange oil. The crude substance was purified by flash column chro­ma­tography on silica gel (15 g) using 20–60% ethyl acetate in hexa­nes as the eluent to yield a yellow solid (0.720 g). The solid was recrystallized from ethanol solution to give a pale-yellow solid (0.508 g, 36.4% over two steps). mp 373–383 K; IR: cm^−1^ 1671.1 (C=O); ^1^H NMR (CDCl_3_): 8.08–7.46 (4H, *m*, aromatics), 6.25 (1H, C2), 3.94 (1H, *tt*, *J* = 12.2 Hz, and *J* = 3.6 Hz, NCH), 3.76 (1H, *dd*, C5, *J* = 0.7 Hz, and *J* = 15.7 Hz), 3.47 (1H, *d*, C5, *J* = 15.7 Hz), 1.96–0.86 (10H, m, cyclo­hexyls); ^13^C NMR: 172.95 (C4), 146.05, 139.12, 134.02, 129.04, 126.72, 125.68, 58.82 (C2), 55.74, 32.20(C5), 31.25, 30.29, 25.89, 25.70, 25.19; MS: (*m*/*z*) 306 (*M* ^+^) C_15_H_18_O_3_N_2_S (306.10).

Crystals for X-ray data collection were grown by dissolving 0.101 g of the solid in hot ethanol and slow evaporation of the solvent.

**1-Cyclo­hexyl-2-(2-nitro­phen­yl)-1,3-thia­zolidin-4-one 1,1-dioxide:** 1-Cyclo­hexyl-2-(2-nitro­phen­yl)-1,3-thia­zolidin-4-one (0.553 mmol) was dissolved in glacial acetic acid (2.4 ml), to which an aqueous solution of KMnO_4_ (175 mg, 1.11 mmol, in 3.0 ml water) was added dropwise at room temperature with vigorous stirring, and stirred for an additional 5 min. Solid sodium bis­ulfite (NaHSO_3_/Na_2_S_2_O_5_) was then added until the solution remained colorless; 3.0 ml of water was then added and the mixture was stirred for a further 10 min. The resulting solid precipitate was filtered and rinsed with water. The resulting powder was purified by recrystallization from CH_3_OH solution. Yield (64%); m.p. 471–472 K; IR: cm^−1^ 1689.6 (C=O), 1326.9, 1308.1, 1162.7 (S=O); ^1^H NMR (CDCl_3_): 8.38 (1H, *dd*, *J* = 8.0, and J = 1.2 Hz, aromatic), 7.78 (1H, *dddd*, *J* = 8.0, 8.0, 1.2, 0.8 Hz, aromatic), 7.68 (1H, *ddd*, *J* = 8.0, 8.0, 1.2 Hz, aromatic), 7.54 (1H, *dd*, *J* = 7.6, 1.2 Hz, aromatic), 6.77 (1H, *s*, C2), 4.41 (1H, *tt*, *J* = 12.0, and *J* = 3.6 Hz, NCH), 3.76 (*dd*, *J* = 16.0 Hz, and *J* = 0.4 Hz, 1H), 3.69 (*d*, *J* = 16.4 Hz, 1 H), 1.96–0.82 (10 H, *m*, cyclo­hexyls); ^13^C NMR: 163.41 (C4), 147.80, 134.43, 131.22, 128.82, 126.92 75.77, 54.52, 50.16, 31.39, 29.67, 25.50, 25.16, 24.84; MS: (*m*/*z*) 339 ([*M* + H]^+^) C_15_H_18_O_5_N_2_S (338.09).

Crystals for X-ray data collection were grown by slow evaporation of a hot methanol solution of the compound.

## Refinement   

Crystal data, data collection and structure refinement details are summarized in Table 3 ▸. The H atoms were placed geometrically and allowed to ride on their parent C atoms during refinement, with C—H distances of 0.93 Å (aromatic), 0.97 Å (methyl­ene) and 0.98 Å (meth­yl), with *U*_iso_(H) = 1.2*U*_eq_(aromatic or methyl­ene C) or 1.5*U*_eq_(methyl C).

## Supplementary Material

Crystal structure: contains datablock(s) 1, 2. DOI: 10.1107/S2056989018015098/hb7781sup1.cif

Structure factors: contains datablock(s) 1. DOI: 10.1107/S2056989018015098/hb77811sup2.hkl

Structure factors: contains datablock(s) 2. DOI: 10.1107/S2056989018015098/hb77812sup3.hkl

Supporting information file. DOI: 10.1107/S2056989018015098/hb77811sup4.cml

Supporting information file. DOI: 10.1107/S2056989018015098/hb77812sup5.cml

CCDC references: 1875395, 1875394

Additional supporting information:  crystallographic information; 3D view; checkCIF report

## Figures and Tables

**Figure 2:**
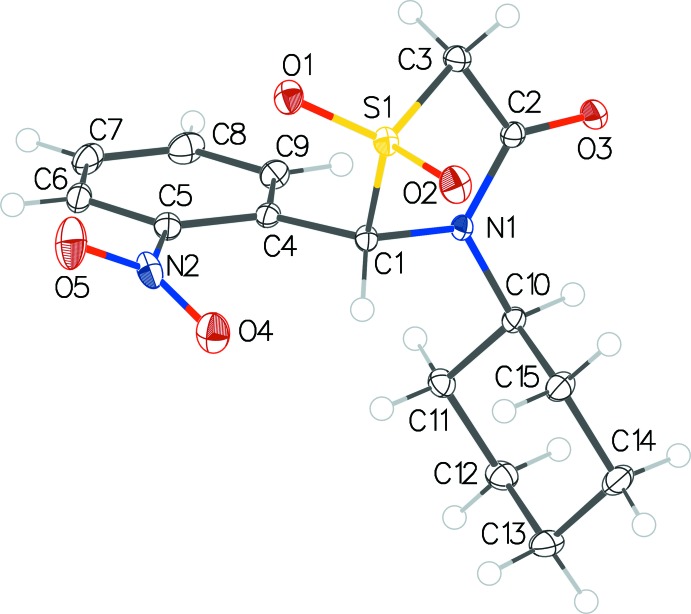
The mol­ecular structure of **2** with displacement ellipsoids drawn at the 50% probability level.

**Table 1:** Hydrogen-bond geometry (Å, °) for **1** [Chem scheme1]

*D*—H⋯*A*	*D*—H	H⋯*A*	*D*⋯*A*	*D*—H⋯*A*
C8—H8⋯O1^i^	0.93	2.65	3.411 (5)	140
C15—H15*B*⋯O3^ii^	0.97	2.66	3.437 (5)	138

Symmetry codes: (i) 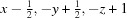; (ii) 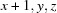.

**Table 2:** Hydrogen-bond geometry (Å, °) for **2** [Chem scheme1]

*D*—H⋯*A*	*D*—H	H⋯*A*	*D*⋯*A*	*D*—H⋯*A*
C3—H3*A*⋯O1^i^	0.99	2.51	3.4594 (16)	161
C3—H3*B*⋯O3^ii^	0.99	2.37	3.3068 (16)	157
C9—H9⋯O1^iii^	0.95	2.80	3.5144 (16)	133
C10—H10⋯O2^iii^	1.00	2.72	3.4381 (16)	129

Symmetry codes: (i) 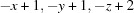; (ii) 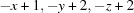; (iii) 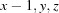.

**Table 3:** Experimental details

	**1**	**2**
Crystal data		
Chemical formula	C_15_H_18_N_2_O_3_S	C_15_H_18_N_2_O_5_S
*M*_r_	306.37	338.37
Crystal system, space group	Orthorhombic, *P* *b* *c* *a*	Triclinic, *P* 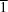
Temperature (K)	298	100
*a*, *b*, *c* (Å)	9.582 (13), 11.444 (15), 26.69 (4)	7.114 (2), 9.401 (3), 12.038 (3)
α, β, γ (°)	90, 90, 90	94.808 (5), 92.110 (5), 107.198 (5)
*V* (Å^3^)	2927 (7)	764.8 (4)
*Z*	8	2
Radiation type	Mo *K*α	Mo *K*α
μ (mm^−1^)	0.23	0.24
Crystal size (mm)	0.27 × 0.25 × 0.2	0.29 × 0.11 × 0.06

Data collection		
Diffractometer	Bruker SMART CCD area detector	Bruker SMART CCD area detector
Absorption correction	Multi-scan (*SADABS*; Bruker, 2001 ▸)	Multi-scan (*SADABS*; Bruker, 2013 ▸)
*T*_min_, *T*_max_	0.732, 0.955	0.815, 0.989
No. of measured, independent and observed [*I* > 2σ(*I*)] reflections	24930, 3685, 2924	9076, 3728, 3475
*R*_int_	0.029	0.015
(sin θ/λ)_max_ (Å^−1^)	0.673	0.663

Refinement		
*R*[*F* ^2^ > 2σ(*F* ^2^)], *wR*(*F* ^2^), *S*	0.053, 0.135, 1.08	0.030, 0.082, 1.05
No. of reflections	3685	3728
No. of parameters	190	208
H-atom treatment	H-atom parameters constrained	H-atom parameters constrained
Δρ_max_, Δρ_min_ (e Å^−3^)	0.35, −0.17	0.45, −0.34

Computer programs: *SMART* and *SAINT* (Bruker, 2001 ▸), *COSMO* and *SAINT* (Bruker, 2013 ▸), *SHELXS97* (Sheldrick, 2008 ▸), *SHELXL2014* and *SHELXL2016* (Sheldrick, 2015 ▸) and *OLEX2* (Dolomanov *et al.*, 2009 ▸).

## supplementary crystallographic information

### 1-Cyclohexyl-2-(2-nitrophenyl)-1,3-thiazolidin-4-one (1) Crystal data

**Table:**

C_15_H_18_N_2_O_3_S	*D*_x_ = 1.391 Mg m^−^^3^
*M**_r_* = 306.37	Mo *K*α radiation, λ = 0.71073 Å
Orthorhombic, *P**b**c**a*	Cell parameters from 5287 reflections
*a* = 9.582 (13) Å	θ = 2.6–24.3°
*b* = 11.444 (15) Å	µ = 0.23 mm^−^^1^
*c* = 26.69 (4) Å	*T* = 298 K
*V* = 2927 (7) Å^3^	Plate, colorless
*Z* = 8	0.27 × 0.25 × 0.2 mm
*F*(000) = 1296	

### 1-Cyclohexyl-2-(2-nitrophenyl)-1,3-thiazolidin-4-one (1) Data collection

**Table:**

Bruker SMART CCD area detector diffractometer	2924 reflections with *I* > 2σ(*I*)
Graphite monochromator	*R*_int_ = 0.029
phi and ω scans	θ_max_ = 28.6°, θ_min_ = 2.6°
Absorption correction: multi-scan (SADABS; Bruker, 2001)	*h* = −12→12
*T*_min_ = 0.732, *T*_max_ = 0.955	*k* = −13→15
24930 measured reflections	*l* = −34→35
3685 independent reflections	

### 1-Cyclohexyl-2-(2-nitrophenyl)-1,3-thiazolidin-4-one (1) Refinement

**Table:**

Refinement on *F*^2^	0 restraints
Least-squares matrix: full	Hydrogen site location: inferred from neighbouring sites
*R*[*F*^2^ > 2σ(*F*^2^)] = 0.053	H-atom parameters constrained
*wR*(*F*^2^) = 0.135	*w* = 1/[σ^2^(*F*_o_^2^) + (0.0614*P*)^2^ + 0.9885*P*] where *P* = (*F*_o_^2^ + 2*F*_c_^2^)/3
*S* = 1.08	(Δ/σ)_max_ = 0.001
3685 reflections	Δρ_max_ = 0.35 e Å^−^^3^
190 parameters	Δρ_min_ = −0.17 e Å^−^^3^

### 1-Cyclohexyl-2-(2-nitrophenyl)-1,3-thiazolidin-4-one (1) Special details

**Table:**

Geometry. All esds (except the esd in the dihedral angle between two l.s. planes) are estimated using the full covariance matrix. The cell esds are taken into account individually in the estimation of esds in distances, angles and torsion angles; correlations between esds in cell parameters are only used when they are defined by crystal symmetry. An approximate (isotropic) treatment of cell esds is used for estimating esds involving l.s. planes.

### 1-Cyclohexyl-2-(2-nitrophenyl)-1,3-thiazolidin-4-one (1) Fractional atomic coordinates and isotropic or equivalent isotropic displacement parameters (Å^2^)

**Table:**

	*x*	*y*	*z*	*U*_iso_*/*U*_eq_	
S1	0.64305 (5)	0.59741 (4)	0.55845 (2)	0.05141 (17)	
O1	0.95808 (13)	0.40036 (13)	0.55305 (5)	0.0543 (4)	
O2	0.43309 (16)	0.62101 (16)	0.64290 (7)	0.0743 (5)	
O3	0.23757 (18)	0.55133 (18)	0.66333 (8)	0.0905 (6)	
N1	0.76951 (13)	0.42423 (13)	0.60346 (5)	0.0360 (3)	
N2	0.34792 (16)	0.54315 (17)	0.64206 (6)	0.0524 (4)	
C1	0.63847 (16)	0.48476 (15)	0.60757 (7)	0.0374 (4)	
H1	0.631265	0.521843	0.640564	0.045*	
C2	0.84294 (17)	0.43948 (18)	0.56067 (7)	0.0417 (4)	
C3	0.7632 (2)	0.5110 (2)	0.52278 (7)	0.0533 (5)	
H3A	0.713666	0.460539	0.499625	0.064*	
H3B	0.826129	0.560651	0.503872	0.064*	
C4	0.51274 (17)	0.40812 (15)	0.59855 (6)	0.0371 (4)	
C5	0.37756 (17)	0.43693 (18)	0.61324 (7)	0.0418 (4)	
C6	0.26504 (19)	0.3690 (2)	0.60127 (8)	0.0533 (5)	
H6	0.176194	0.390377	0.611901	0.064*	
C7	0.2834 (2)	0.2698 (2)	0.57371 (8)	0.0603 (6)	
H7	0.206700	0.224720	0.564627	0.072*	
C8	0.4151 (2)	0.2367 (2)	0.55939 (8)	0.0585 (5)	
H8	0.428365	0.168290	0.541158	0.070*	
C9	0.5272 (2)	0.30517 (18)	0.57219 (7)	0.0470 (4)	
H9	0.616167	0.281283	0.562734	0.056*	
C10	0.83474 (16)	0.37054 (16)	0.64788 (6)	0.0359 (4)	
H10	0.911045	0.320833	0.635901	0.043*	
C11	0.73624 (19)	0.29343 (17)	0.67713 (6)	0.0438 (4)	
H11A	0.701899	0.231608	0.655539	0.053*	
H11B	0.656828	0.339271	0.688165	0.053*	
C12	0.8082 (2)	0.2396 (2)	0.72259 (7)	0.0544 (5)	
H12A	0.740381	0.195440	0.741803	0.065*	
H12B	0.879757	0.185814	0.711273	0.065*	
C13	0.8736 (2)	0.3306 (2)	0.75583 (7)	0.0580 (6)	
H13A	0.924527	0.292580	0.782674	0.070*	
H13B	0.801130	0.378481	0.770746	0.070*	
C14	0.9720 (2)	0.4073 (2)	0.72616 (8)	0.0602 (6)	
H14A	1.050130	0.360810	0.714471	0.072*	
H14B	1.008345	0.468373	0.747720	0.072*	
C15	0.89855 (19)	0.46261 (18)	0.68135 (8)	0.0498 (5)	
H15A	0.825973	0.514902	0.693159	0.060*	
H15B	0.965152	0.508361	0.662267	0.060*	

### 1-Cyclohexyl-2-(2-nitrophenyl)-1,3-thiazolidin-4-one (1) Atomic displacement parameters (Å^2^)

**Table:**

	*U*^11^	*U*^22^	*U*^33^	*U*^12^	*U*^13^	*U*^23^
S1	0.0457 (3)	0.0450 (3)	0.0635 (3)	0.0006 (2)	−0.0051 (2)	0.0116 (2)
O1	0.0367 (7)	0.0788 (10)	0.0473 (8)	0.0056 (6)	0.0121 (5)	0.0074 (7)
O2	0.0446 (8)	0.0696 (11)	0.1087 (14)	0.0105 (8)	−0.0022 (8)	−0.0240 (10)
O3	0.0650 (10)	0.0923 (14)	0.1142 (15)	0.0137 (10)	0.0447 (11)	−0.0008 (12)
N1	0.0265 (6)	0.0465 (8)	0.0350 (7)	0.0030 (6)	0.0009 (5)	0.0024 (6)
N2	0.0391 (8)	0.0655 (11)	0.0525 (10)	0.0153 (8)	0.0033 (7)	0.0072 (8)
C1	0.0293 (7)	0.0437 (9)	0.0392 (9)	0.0029 (7)	−0.0013 (6)	0.0000 (7)
C2	0.0358 (8)	0.0510 (10)	0.0384 (9)	−0.0043 (7)	0.0015 (7)	0.0026 (8)
C3	0.0499 (10)	0.0674 (13)	0.0427 (10)	−0.0001 (10)	0.0020 (8)	0.0133 (9)
C4	0.0297 (7)	0.0488 (10)	0.0328 (8)	0.0009 (7)	−0.0028 (6)	0.0050 (7)
C5	0.0335 (8)	0.0559 (11)	0.0361 (9)	0.0031 (7)	−0.0018 (7)	0.0113 (8)
C6	0.0319 (8)	0.0767 (15)	0.0514 (11)	−0.0066 (9)	−0.0026 (8)	0.0171 (10)
C7	0.0471 (11)	0.0782 (16)	0.0557 (12)	−0.0246 (11)	−0.0119 (9)	0.0133 (12)
C8	0.0618 (13)	0.0614 (13)	0.0522 (12)	−0.0143 (11)	−0.0078 (10)	−0.0044 (10)
C9	0.0401 (9)	0.0551 (11)	0.0458 (10)	−0.0031 (8)	−0.0012 (8)	−0.0047 (9)
C10	0.0277 (7)	0.0446 (9)	0.0354 (8)	0.0052 (6)	0.0010 (6)	0.0004 (7)
C11	0.0447 (9)	0.0524 (11)	0.0341 (9)	−0.0078 (8)	−0.0021 (7)	−0.0002 (8)
C12	0.0598 (11)	0.0640 (13)	0.0395 (10)	−0.0026 (10)	−0.0014 (9)	0.0090 (9)
C13	0.0509 (11)	0.0862 (16)	0.0368 (10)	0.0024 (11)	−0.0085 (8)	−0.0009 (10)
C14	0.0443 (10)	0.0800 (16)	0.0564 (12)	−0.0056 (10)	−0.0177 (9)	−0.0020 (11)
C15	0.0384 (9)	0.0538 (12)	0.0570 (12)	−0.0061 (8)	−0.0118 (8)	−0.0006 (9)

### 1-Cyclohexyl-2-(2-nitrophenyl)-1,3-thiazolidin-4-one (1) Geometric parameters (Å, º)

**Table:**

S1—C1	1.839 (3)	C8—H8	0.9300
S1—C3	1.792 (3)	C8—C9	1.373 (3)
O1—C2	1.208 (2)	C9—H9	0.9300
O2—N2	1.209 (3)	C10—H10	0.9800
O3—N2	1.204 (2)	C10—C11	1.510 (3)
N1—C1	1.438 (3)	C10—C15	1.511 (3)
N1—C2	1.353 (3)	C11—H11A	0.9700
N1—C10	1.474 (2)	C11—H11B	0.9700
N2—C5	1.466 (3)	C11—C12	1.525 (3)
C1—H1	0.9800	C12—H12A	0.9700
C1—C4	1.509 (3)	C12—H12B	0.9700
C2—C3	1.508 (3)	C12—C13	1.505 (3)
C3—H3A	0.9700	C13—H13A	0.9700
C3—H3B	0.9700	C13—H13B	0.9700
C4—C5	1.393 (3)	C13—C14	1.511 (3)
C4—C9	1.379 (3)	C14—H14A	0.9700
C5—C6	1.367 (3)	C14—H14B	0.9700
C6—H6	0.9300	C14—C15	1.525 (3)
C6—C7	1.364 (4)	C15—H15A	0.9700
C7—H7	0.9300	C15—H15B	0.9700
C7—C8	1.372 (4)		
			
C3—S1—C1	90.40 (12)	C8—C9—C4	122.42 (19)
C1—N1—C10	120.64 (14)	C8—C9—H9	118.8
C2—N1—C1	117.15 (15)	N1—C10—H10	107.2
C2—N1—C10	120.80 (16)	N1—C10—C11	113.22 (15)
O2—N2—C5	119.36 (17)	N1—C10—C15	110.87 (17)
O3—N2—O2	121.8 (2)	C11—C10—H10	107.2
O3—N2—C5	118.8 (2)	C11—C10—C15	110.80 (17)
S1—C1—H1	109.8	C15—C10—H10	107.2
N1—C1—S1	105.22 (12)	C10—C11—H11A	109.4
N1—C1—H1	109.8	C10—C11—H11B	109.4
N1—C1—C4	113.88 (17)	C10—C11—C12	111.39 (17)
C4—C1—S1	108.22 (12)	H11A—C11—H11B	108.0
C4—C1—H1	109.8	C12—C11—H11A	109.4
O1—C2—N1	124.72 (17)	C12—C11—H11B	109.4
O1—C2—C3	123.40 (17)	C11—C12—H12A	109.2
N1—C2—C3	111.88 (17)	C11—C12—H12B	109.2
S1—C3—H3A	110.6	H12A—C12—H12B	107.9
S1—C3—H3B	110.6	C13—C12—C11	112.2 (2)
C2—C3—S1	105.59 (16)	C13—C12—H12A	109.2
C2—C3—H3A	110.6	C13—C12—H12B	109.2
C2—C3—H3B	110.6	C12—C13—H13A	109.5
H3A—C3—H3B	108.8	C12—C13—H13B	109.5
C5—C4—C1	124.04 (18)	C12—C13—C14	110.66 (19)
C9—C4—C1	119.83 (16)	H13A—C13—H13B	108.1
C9—C4—C5	116.05 (17)	C14—C13—H13A	109.5
C4—C5—N2	121.60 (17)	C14—C13—H13B	109.5
C6—C5—N2	116.18 (18)	C13—C14—H14A	109.4
C6—C5—C4	122.2 (2)	C13—C14—H14B	109.4
C5—C6—H6	120.1	C13—C14—C15	111.34 (18)
C7—C6—C5	119.84 (19)	H14A—C14—H14B	108.0
C7—C6—H6	120.1	C15—C14—H14A	109.4
C6—C7—H7	120.0	C15—C14—H14B	109.4
C6—C7—C8	119.92 (19)	C10—C15—C14	111.15 (19)
C8—C7—H7	120.0	C10—C15—H15A	109.4
C7—C8—H8	120.3	C10—C15—H15B	109.4
C7—C8—C9	119.5 (2)	C14—C15—H15A	109.4
C9—C8—H8	120.3	C14—C15—H15B	109.4
C4—C9—H9	118.8	H15A—C15—H15B	108.0
			
S1—C1—C4—C5	−81.4 (2)	C2—N1—C1—C4	102.13 (19)
S1—C1—C4—C9	95.38 (19)	C2—N1—C10—C11	−143.59 (18)
O1—C2—C3—S1	−155.76 (17)	C2—N1—C10—C15	91.2 (2)
O2—N2—C5—C4	18.2 (3)	C3—S1—C1—N1	25.72 (13)
O2—N2—C5—C6	−161.08 (19)	C3—S1—C1—C4	−96.37 (16)
O3—N2—C5—C4	−163.46 (19)	C4—C5—C6—C7	−0.5 (3)
O3—N2—C5—C6	17.2 (3)	C5—C4—C9—C8	2.3 (3)
N1—C1—C4—C5	162.03 (16)	C5—C6—C7—C8	2.0 (3)
N1—C1—C4—C9	−21.2 (2)	C6—C7—C8—C9	−1.3 (3)
N1—C2—C3—S1	24.8 (2)	C7—C8—C9—C4	−1.0 (3)
N1—C10—C11—C12	179.86 (15)	C9—C4—C5—N2	179.17 (16)
N1—C10—C15—C14	−177.41 (15)	C9—C4—C5—C6	−1.6 (3)
N2—C5—C6—C7	178.77 (17)	C10—N1—C1—S1	150.33 (13)
C1—S1—C3—C2	−28.32 (15)	C10—N1—C1—C4	−91.31 (19)
C1—N1—C2—O1	175.24 (18)	C10—N1—C2—O1	8.7 (3)
C1—N1—C2—C3	−5.3 (2)	C10—N1—C2—C3	−171.82 (16)
C1—N1—C10—C11	50.3 (2)	C10—C11—C12—C13	54.7 (2)
C1—N1—C10—C15	−74.9 (2)	C11—C10—C15—C14	56.0 (2)
C1—C4—C5—N2	−4.0 (3)	C11—C12—C13—C14	−54.6 (2)
C1—C4—C5—C6	175.29 (17)	C12—C13—C14—C15	55.4 (3)
C1—C4—C9—C8	−174.68 (18)	C13—C14—C15—C10	−56.6 (2)
C2—N1—C1—S1	−16.23 (18)	C15—C10—C11—C12	−54.8 (2)

### 1-Cyclohexyl-2-(2-nitrophenyl)-1,3-thiazolidin-4-one (1) Hydrogen-bond geometry (Å, º)

**Table:**

*D*—H···*A*	*D*—H	H···*A*	*D*···*A*	*D*—H···*A*
C8—H8···O1^i^	0.93	2.65	3.411 (5)	140
C15—H15*B*···O3^ii^	0.97	2.66	3.437 (5)	138

Symmetry codes: (i) *x*−1/2, −*y*+1/2, −*z*+1; (ii) *x*+1, *y*, *z*.

### 1-Cyclohexyl-2-(2-nitrophenyl)-1,3-thiazolidin-4-one 1,1-dioxide (2) Crystal data

**Table:**

C_15_H_18_N_2_O_5_S	*Z* = 2
*M**_r_* = 338.37	*F*(000) = 356
Triclinic, *P*1	*D*_x_ = 1.469 Mg m^−^^3^
*a* = 7.114 (2) Å	Mo *K*α radiation, λ = 0.71073 Å
*b* = 9.401 (3) Å	Cell parameters from 1021 reflections
*c* = 12.038 (3) Å	θ = 2.6–25.0°
α = 94.808 (5)°	µ = 0.24 mm^−^^1^
β = 92.110 (5)°	*T* = 100 K
γ = 107.198 (5)°	Block, colorless
*V* = 764.8 (4) Å^3^	0.29 × 0.11 × 0.06 mm

### 1-Cyclohexyl-2-(2-nitrophenyl)-1,3-thiazolidin-4-one 1,1-dioxide (2) Data collection

**Table:**

Bruker SMART CCD area detector diffractometer	3728 independent reflections
Radiation source: fine-focus sealed tube	3475 reflections with *I* > 2σ(*I*)
Graphite monochromator	*R*_int_ = 0.015
phi and ω scans	θ_max_ = 28.1°, θ_min_ = 1.7°
Absorption correction: multi-scan (SADABS; Bruker, 2013)	*h* = −9→9
*T*_min_ = 0.815, *T*_max_ = 0.989	*k* = −12→12
9076 measured reflections	*l* = −15→15

### 1-Cyclohexyl-2-(2-nitrophenyl)-1,3-thiazolidin-4-one 1,1-dioxide (2) Refinement

**Table:**

Refinement on *F*^2^	Primary atom site location: structure-invariant direct methods
Least-squares matrix: full	Secondary atom site location: difference Fourier map
*R*[*F*^2^ > 2σ(*F*^2^)] = 0.030	Hydrogen site location: inferred from neighbouring sites
*wR*(*F*^2^) = 0.082	H-atom parameters constrained
*S* = 1.05	*w* = 1/[σ^2^(*F*_o_^2^) + (0.0404*P*)^2^ + 0.4077*P*] where *P* = (*F*_o_^2^ + 2*F*_c_^2^)/3
3728 reflections	(Δ/σ)_max_ = 0.001
208 parameters	Δρ_max_ = 0.45 e Å^−^^3^
0 restraints	Δρ_min_ = −0.34 e Å^−^^3^

### 1-Cyclohexyl-2-(2-nitrophenyl)-1,3-thiazolidin-4-one 1,1-dioxide (2) Special details

**Table:**

Geometry. All esds (except the esd in the dihedral angle between two l.s. planes) are estimated using the full covariance matrix. The cell esds are taken into account individually in the estimation of esds in distances, angles and torsion angles; correlations between esds in cell parameters are only used when they are defined by crystal symmetry. An approximate (isotropic) treatment of cell esds is used for estimating esds involving l.s. planes.
Refinement. Refinement of F^2^ against ALL reflections. The weighted R-factor wR and goodness of fit S are based on F^2^, conventional R-factors R are based on F, with F set to zero for negative F^2^. The threshold expression of F^2^ > 2sigma(F^2^) is used only for calculating R-factors(gt) etc. and is not relevant to the choice of reflections for refinement. R-factors based on F^2^ are statistically about twice as large as those based on F, and R- factors based on ALL data will be even larger.

### 1-Cyclohexyl-2-(2-nitrophenyl)-1,3-thiazolidin-4-one 1,1-dioxide (2) Fractional atomic coordinates and isotropic or equivalent isotropic displacement parameters (Å^2^)

**Table:**

	*x*	*y*	*z*	*U*_iso_*/*U*_eq_	
S1	0.60965 (4)	0.67214 (3)	0.85449 (2)	0.01180 (8)	
O1	0.67190 (12)	0.55058 (9)	0.89218 (7)	0.01731 (18)	
O2	0.74722 (12)	0.78778 (9)	0.80246 (8)	0.01862 (18)	
O3	0.27805 (12)	0.90586 (9)	0.92007 (7)	0.01539 (17)	
O4	0.54722 (14)	0.41291 (10)	0.63921 (8)	0.0228 (2)	
O5	0.57817 (15)	0.22722 (11)	0.72569 (10)	0.0296 (2)	
N1	0.29029 (13)	0.71982 (10)	0.78749 (8)	0.01082 (18)	
N2	0.49472 (15)	0.32058 (11)	0.70700 (9)	0.0170 (2)	
C1	0.38198 (15)	0.60257 (11)	0.76329 (9)	0.0105 (2)	
H1	0.4147	0.5983	0.6832	0.013*	
C2	0.34073 (16)	0.80345 (12)	0.88860 (9)	0.0114 (2)	
C3	0.49252 (16)	0.75554 (12)	0.95740 (9)	0.0136 (2)	
H3A	0.4276	0.6827	1.0094	0.016*	
H3B	0.5881	0.8429	1.0007	0.016*	
C4	0.26158 (16)	0.44866 (12)	0.79064 (9)	0.0112 (2)	
C5	0.31876 (16)	0.31896 (12)	0.76822 (9)	0.0130 (2)	
C6	0.21380 (18)	0.18142 (13)	0.80081 (10)	0.0166 (2)	
H6	0.2592	0.0968	0.7858	0.020*	
C7	0.04245 (19)	0.16798 (14)	0.85541 (11)	0.0198 (2)	
H7	−0.0318	0.0740	0.8773	0.024*	
C8	−0.01917 (18)	0.29359 (14)	0.87772 (10)	0.0193 (2)	
H8	−0.1375	0.2850	0.9143	0.023*	
C9	0.08976 (17)	0.43194 (13)	0.84728 (10)	0.0146 (2)	
H9	0.0464	0.5169	0.8654	0.018*	
C10	0.18316 (16)	0.76881 (12)	0.69763 (9)	0.0117 (2)	
H10	0.1191	0.8408	0.7335	0.014*	
C11	0.02037 (16)	0.63935 (12)	0.63537 (9)	0.0137 (2)	
H11A	−0.0743	0.5895	0.6886	0.016*	
H11B	0.0783	0.5647	0.5997	0.016*	
C12	−0.08705 (17)	0.69932 (13)	0.54591 (10)	0.0167 (2)	
H12A	−0.1903	0.6151	0.5043	0.020*	
H12B	−0.1522	0.7688	0.5824	0.020*	
C13	0.05666 (19)	0.78087 (14)	0.46475 (10)	0.0190 (2)	
H13A	−0.0150	0.8207	0.4090	0.023*	
H13B	0.1151	0.7098	0.4244	0.023*	
C14	0.22021 (18)	0.90929 (13)	0.52708 (10)	0.0188 (2)	
H14A	0.1627	0.9848	0.5618	0.023*	
H14B	0.3150	0.9582	0.4735	0.023*	
C15	0.32904 (17)	0.85282 (13)	0.61788 (10)	0.0156 (2)	
H15A	0.3984	0.7854	0.5827	0.019*	
H15B	0.4287	0.9387	0.6602	0.019*	

### 1-Cyclohexyl-2-(2-nitrophenyl)-1,3-thiazolidin-4-one 1,1-dioxide (2) Atomic displacement parameters (Å^2^)

**Table:**

	*U*^11^	*U*^22^	*U*^33^	*U*^12^	*U*^13^	*U*^23^
S1	0.00952 (13)	0.01062 (13)	0.01482 (14)	0.00278 (9)	−0.00059 (9)	0.00021 (9)
O1	0.0150 (4)	0.0156 (4)	0.0222 (4)	0.0069 (3)	−0.0040 (3)	0.0006 (3)
O2	0.0134 (4)	0.0156 (4)	0.0239 (4)	−0.0002 (3)	0.0037 (3)	0.0016 (3)
O3	0.0187 (4)	0.0137 (4)	0.0148 (4)	0.0072 (3)	0.0002 (3)	−0.0008 (3)
O4	0.0229 (4)	0.0203 (4)	0.0274 (5)	0.0086 (4)	0.0096 (4)	0.0034 (4)
O5	0.0233 (5)	0.0216 (5)	0.0491 (6)	0.0149 (4)	0.0014 (4)	0.0033 (4)
N1	0.0128 (4)	0.0093 (4)	0.0114 (4)	0.0050 (3)	−0.0005 (3)	0.0008 (3)
N2	0.0142 (4)	0.0121 (4)	0.0240 (5)	0.0047 (4)	−0.0021 (4)	−0.0039 (4)
C1	0.0104 (4)	0.0090 (5)	0.0123 (5)	0.0033 (4)	0.0000 (4)	0.0008 (4)
C2	0.0116 (5)	0.0102 (5)	0.0115 (5)	0.0015 (4)	0.0010 (4)	0.0024 (4)
C3	0.0154 (5)	0.0142 (5)	0.0119 (5)	0.0062 (4)	−0.0010 (4)	−0.0001 (4)
C4	0.0109 (5)	0.0104 (5)	0.0115 (5)	0.0022 (4)	−0.0022 (4)	0.0014 (4)
C5	0.0116 (5)	0.0121 (5)	0.0144 (5)	0.0031 (4)	−0.0030 (4)	0.0003 (4)
C6	0.0201 (5)	0.0104 (5)	0.0179 (5)	0.0030 (4)	−0.0065 (4)	0.0017 (4)
C7	0.0213 (6)	0.0147 (5)	0.0190 (6)	−0.0023 (4)	−0.0036 (4)	0.0065 (4)
C8	0.0160 (5)	0.0221 (6)	0.0176 (6)	0.0010 (4)	0.0028 (4)	0.0062 (5)
C9	0.0143 (5)	0.0151 (5)	0.0147 (5)	0.0047 (4)	0.0007 (4)	0.0022 (4)
C10	0.0131 (5)	0.0104 (5)	0.0122 (5)	0.0046 (4)	−0.0015 (4)	0.0015 (4)
C11	0.0130 (5)	0.0122 (5)	0.0145 (5)	0.0025 (4)	−0.0009 (4)	−0.0002 (4)
C12	0.0153 (5)	0.0189 (5)	0.0156 (5)	0.0062 (4)	−0.0030 (4)	−0.0011 (4)
C13	0.0228 (6)	0.0224 (6)	0.0130 (5)	0.0089 (5)	−0.0028 (4)	0.0022 (4)
C14	0.0224 (6)	0.0173 (5)	0.0169 (5)	0.0052 (5)	−0.0017 (4)	0.0067 (4)
C15	0.0155 (5)	0.0147 (5)	0.0160 (5)	0.0029 (4)	−0.0007 (4)	0.0047 (4)

### 1-Cyclohexyl-2-(2-nitrophenyl)-1,3-thiazolidin-4-one 1,1-dioxide (2) Geometric parameters (Å, º)

**Table:**

S1—O1	1.4419 (9)	C7—C8	1.3858 (18)
S1—O2	1.4360 (9)	C8—H8	0.9500
S1—C1	1.8382 (12)	C8—C9	1.3896 (16)
S1—C3	1.7729 (12)	C9—H9	0.9500
O3—C2	1.2143 (14)	C10—H10	1.0000
O4—N2	1.2281 (14)	C10—C11	1.5276 (15)
O5—N2	1.2267 (14)	C10—C15	1.5291 (16)
N1—C1	1.4527 (13)	C11—H11A	0.9900
N1—C2	1.3671 (14)	C11—H11B	0.9900
N1—C10	1.4810 (14)	C11—C12	1.5354 (16)
N2—C5	1.4726 (15)	C12—H12A	0.9900
C1—H1	1.0000	C12—H12B	0.9900
C1—C4	1.5177 (15)	C12—C13	1.5242 (17)
C2—C3	1.5294 (15)	C13—H13A	0.9900
C3—H3A	0.9900	C13—H13B	0.9900
C3—H3B	0.9900	C13—C14	1.5257 (17)
C4—C5	1.4040 (15)	C14—H14A	0.9900
C4—C9	1.3963 (16)	C14—H14B	0.9900
C5—C6	1.3853 (16)	C14—C15	1.5342 (16)
C6—H6	0.9500	C15—H15A	0.9900
C6—C7	1.3841 (18)	C15—H15B	0.9900
C7—H7	0.9500		
			
O1—S1—C1	111.35 (5)	C9—C8—H8	119.5
O1—S1—C3	113.14 (6)	C4—C9—H9	119.3
O2—S1—O1	119.26 (6)	C8—C9—C4	121.39 (11)
O2—S1—C1	108.34 (5)	C8—C9—H9	119.3
O2—S1—C3	109.11 (6)	N1—C10—H10	107.5
C3—S1—C1	92.26 (5)	N1—C10—C11	112.55 (9)
C1—N1—C10	120.68 (9)	N1—C10—C15	109.90 (9)
C2—N1—C1	117.45 (9)	C11—C10—H10	107.5
C2—N1—C10	120.48 (9)	C11—C10—C15	111.61 (9)
O4—N2—C5	118.45 (10)	C15—C10—H10	107.5
O5—N2—O4	123.92 (11)	C10—C11—H11A	109.8
O5—N2—C5	117.62 (11)	C10—C11—H11B	109.8
S1—C1—H1	110.0	C10—C11—C12	109.49 (9)
N1—C1—S1	101.36 (7)	H11A—C11—H11B	108.2
N1—C1—H1	110.0	C12—C11—H11A	109.8
N1—C1—C4	114.61 (9)	C12—C11—H11B	109.8
C4—C1—S1	110.64 (7)	C11—C12—H12A	109.5
C4—C1—H1	110.0	C11—C12—H12B	109.5
O3—C2—N1	124.96 (10)	H12A—C12—H12B	108.1
O3—C2—C3	123.40 (10)	C13—C12—C11	110.88 (10)
N1—C2—C3	111.61 (9)	C13—C12—H12A	109.5
S1—C3—H3A	111.1	C13—C12—H12B	109.5
S1—C3—H3B	111.1	C12—C13—H13A	109.5
C2—C3—S1	103.22 (8)	C12—C13—H13B	109.5
C2—C3—H3A	111.1	C12—C13—C14	110.58 (10)
C2—C3—H3B	111.1	H13A—C13—H13B	108.1
H3A—C3—H3B	109.1	C14—C13—H13A	109.5
C5—C4—C1	123.62 (10)	C14—C13—H13B	109.5
C9—C4—C1	120.00 (10)	C13—C14—H14A	109.4
C9—C4—C5	116.27 (10)	C13—C14—H14B	109.4
C4—C5—N2	121.73 (10)	C13—C14—C15	111.06 (10)
C6—C5—N2	115.57 (10)	H14A—C14—H14B	108.0
C6—C5—C4	122.70 (11)	C15—C14—H14A	109.4
C5—C6—H6	120.2	C15—C14—H14B	109.4
C7—C6—C5	119.66 (11)	C10—C15—C14	110.26 (10)
C7—C6—H6	120.2	C10—C15—H15A	109.6
C6—C7—H7	120.5	C10—C15—H15B	109.6
C6—C7—C8	119.05 (11)	C14—C15—H15A	109.6
C8—C7—H7	120.5	C14—C15—H15B	109.6
C7—C8—H8	119.5	H15A—C15—H15B	108.1
C7—C8—C9	120.90 (12)		
			
S1—C1—C4—C5	−69.60 (12)	C1—C4—C5—C6	175.39 (10)
S1—C1—C4—C9	106.31 (10)	C1—C4—C9—C8	−177.24 (10)
O1—S1—C1—N1	148.94 (7)	C2—N1—C1—S1	−24.61 (11)
O1—S1—C1—C4	26.96 (9)	C2—N1—C1—C4	94.57 (11)
O1—S1—C3—C2	−147.19 (7)	C2—N1—C10—C11	−138.49 (10)
O2—S1—C1—N1	−78.01 (8)	C2—N1—C10—C15	96.45 (12)
O2—S1—C1—C4	160.01 (7)	C3—S1—C1—N1	32.98 (7)
O2—S1—C3—C2	77.52 (8)	C3—S1—C1—C4	−88.99 (8)
O3—C2—C3—S1	−153.99 (9)	C4—C5—C6—C7	1.63 (17)
O4—N2—C5—C4	−28.26 (16)	C5—C4—C9—C8	−1.03 (16)
O4—N2—C5—C6	151.15 (11)	C5—C6—C7—C8	−0.87 (17)
O5—N2—C5—C4	152.98 (11)	C6—C7—C8—C9	−0.79 (18)
O5—N2—C5—C6	−27.60 (15)	C7—C8—C9—C4	1.78 (18)
N1—C1—C4—C5	176.56 (10)	C9—C4—C5—N2	178.71 (10)
N1—C1—C4—C9	−7.52 (14)	C9—C4—C5—C6	−0.67 (16)
N1—C2—C3—S1	24.31 (11)	C10—N1—C1—S1	141.97 (8)
N1—C10—C11—C12	178.54 (9)	C10—N1—C1—C4	−98.85 (11)
N1—C10—C15—C14	−177.81 (9)	C10—N1—C2—O3	12.62 (16)
N2—C5—C6—C7	−177.78 (10)	C10—N1—C2—C3	−165.64 (9)
C1—S1—C3—C2	−32.79 (8)	C10—C11—C12—C13	57.68 (12)
C1—N1—C2—O3	179.23 (10)	C11—C10—C15—C14	56.59 (12)
C1—N1—C2—C3	0.97 (13)	C11—C12—C13—C14	−57.84 (13)
C1—N1—C10—C11	55.34 (13)	C12—C13—C14—C15	56.73 (13)
C1—N1—C10—C15	−69.72 (12)	C13—C14—C15—C10	−55.78 (13)
C1—C4—C5—N2	−5.23 (16)	C15—C10—C11—C12	−57.33 (12)

### 1-Cyclohexyl-2-(2-nitrophenyl)-1,3-thiazolidin-4-one 1,1-dioxide (2) Hydrogen-bond geometry (Å, º)

**Table:**

*D*—H···*A*	*D*—H	H···*A*	*D*···*A*	*D*—H···*A*
C3—H3*A*···O1^i^	0.99	2.51	3.4594 (16)	161
C3—H3*B*···O3^ii^	0.99	2.37	3.3068 (16)	157
C9—H9···O1^iii^	0.95	2.80	3.5144 (16)	133
C10—H10···O2^iii^	1.00	2.72	3.4381 (16)	129

Symmetry codes: (i) −*x*+1, −*y*+1, −*z*+2; (ii) −*x*+1, −*y*+2, −*z*+2; (iii) *x*−1, *y*, *z*.
